# Secondary Structures of rRNAs from All Three Domains of Life

**DOI:** 10.1371/journal.pone.0088222

**Published:** 2014-02-05

**Authors:** Anton S. Petrov, Chad R. Bernier, Burak Gulen, Chris C. Waterbury, Eli Hershkovits, Chiaolong Hsiao, Stephen C. Harvey, Nicholas V. Hud, George E. Fox, Roger M. Wartell, Loren Dean Williams

**Affiliations:** 1 Center for Ribosomal Origins and Evolution, Georgia Institute of Technology, Atlanta, Georgia, United States of America; 2 School of Chemistry and Biochemistry Georgia Institute of Technology, Atlanta, Georgia, United States of America; 3 Department of Biology and Biochemistry, University of Houston, Houston, Texas, United States of America; Ben-Gurion University, Israel

## Abstract

Accurate secondary structures are important for understanding ribosomes, which are extremely large and highly complex. Using 3D structures of ribosomes as input, we have revised and corrected traditional secondary (2°) structures of rRNAs. We identify helices by specific geometric and molecular interaction criteria, not by co-variation. The structural approach allows us to incorporate non-canonical base pairs on parity with Watson-Crick base pairs. The resulting rRNA 2° structures are up-to-date and consistent with three-dimensional structures, and are information-rich. These 2° structures are relatively simple to understand and are amenable to reproduction and modification by end-users. The 2° structures made available here broadly sample the phylogenetic tree and are mapped with a variety of data related to molecular interactions and geometry, phylogeny and evolution. We have generated 2° structures for both large subunit (LSU) 23S/28S and small subunit (SSU) 16S/18S rRNAs of *Escherichia coli, Thermus thermophilus, Haloarcula marismortui* (LSU rRNA only), *Saccharomyces cerevisiae*, *Drosophila melanogaster*, and *Homo sapiens*. We provide high-resolution editable versions of the 2° structures in several file formats. For the SSU rRNA, the 2° structures use an intuitive representation of the central pseudoknot where base triples are presented as pairs of base pairs. Both LSU and SSU secondary maps are available (http://apollo.chemistry.gatech.edu/RibosomeGallery). Mapping of data onto 2° structures was performed on the RiboVision server (http://apollo.chemistry.gatech.edu/RiboVision).

## Introduction

RNA secondary (2°) structures, with symbolic representations of base pairs, double-helices, loops, bulges, and single-strands, provide frameworks for understanding three-dimensional (3D) structure, folding and function of RNA, and for organizing, distilling, and illustrating a wide variety of information. Accurate and accessible 2° structures are particularly important for understanding ribosomes, which are extremely large and highly complex three-dimensional objects.

Co-variation approaches, using a rich sequence database as primary input, are powerful and widely-applicable for determining rRNA 2° structures in the absence of 3D information. Co-variation methods produce very few false-positive base pairs [1]. However, 2° structures determined by co-variation have inherent limitations. Co-variation does not reliably reveal non-canonical base pairs, especially purine-purine base pairs. For example, Helix 26a of LSU rRNAs was not detected by co-variation methods and was not included in traditional 2° structures [1], [2]. The rRNA comprising Helix 26a is represented by an extended single-strand in co-variation 2° structures. The omission of Helix 26a is significant because it is universally-conserved and thermodynamically stable [3], [4], and is a core component that helps define domain architecture [5].

Here we focus on accurate re-determination of 2° structures, primarily of SSU rRNAs. We modify the traditional *E. coli* SSU 2° structure to incorporate non-canonical base pairs. In addition, we include all base pairing interactions of the central pseudoknot. And finally, for several eukaryotic species, we provide complete 2° structures of both subunits, including expansion segments. Co-variation approaches are especially problematic for highly idiosyncratic RNA sequence regions such as expansion segments, because appropriate sets of alignable sequences may not be available or readily identifiable.

We have constructed 2° structures that minimize artificial fragmentation of rRNA. For historical reasons, 2° structures, especially those of larger rRNAs, are represented as fragments placed around the conserved core. Optimal 2° structures should as far as possible portray the true continuity of an rRNA strand. In practice, representation of rRNA as continuous strands can require re-organizing the traditional scheme of the common core and may not be desirable in all instances. The major differences between the co-variation and 3D based 2° structures are highlighted in Figure S1.

The small but growing number of ribosomal 3D structures allows 2° structure determination by geometric analysis. Information from 3D structures can be used to determine accurate 2° structures, including non-canonical base-pairs and expansion segments. Thus, we have used geometric analysis of 3D structures of ribosomes to re-determine rRNA 2° structures. The resulting 3D based 2° structures, unlike co-variation 2° structures, contain all base pairs and helices observed in 3D structures.

We make available a series of 2° structures that broadly sample the phylogenetic tree, are up-to-date, and as far as possible, accurately represent strand continuity. We have incorporated non-canonical base pairs. We have mapped the 2° structures with a variety of data related to molecular interactions and geometry, phylogeny and evolution. We have partitioned the rRNA into helices and domains. These information-rich 2° structures are amenable to reproduction and modification by end-users. We provide high-resolution editable versions of the 2° structures in several file formats. The images are legible when printed on a single sheet of standard sized paper. Both LSU and SSU secondary maps are available (http://apollo.chemistry.gatech.edu/RibosomeGallery). Mapping of data onto 2° structures was performed on the RiboVision server (http://apollo.chemistry.gatech.edu/RiboVision) [10].

**Figure 1:**
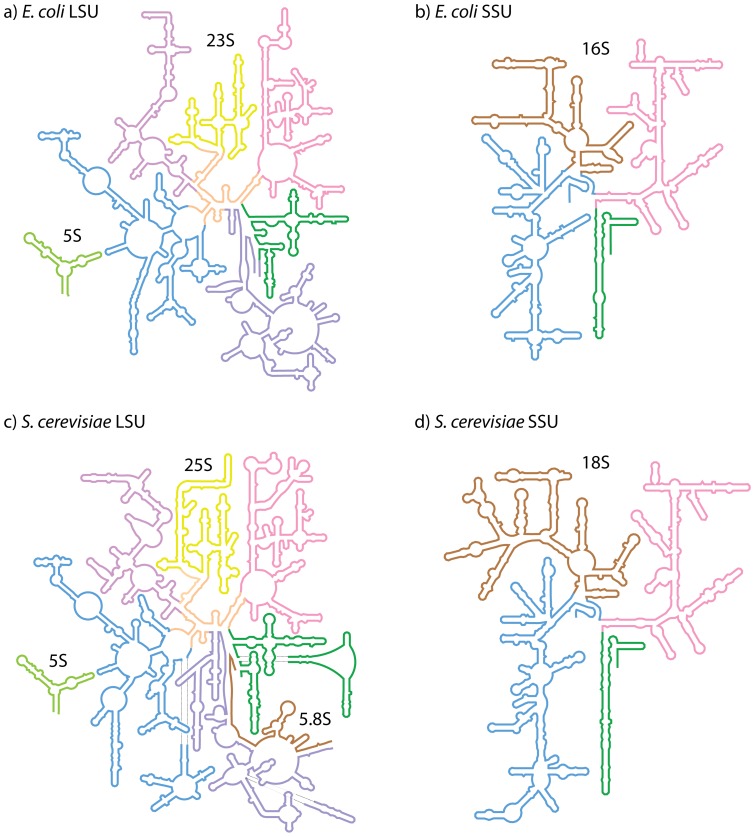
Schematic rRNA 2° structures of a) *E. coli* LSU, b) *E. coli* SSU, c) *S. cerevisiae* LSU, and d) *S. cerevisiae* SSU. These 2° structures are derived from 3D structures, and include non-canonical base pairs. The domain colors in the LSU are, Domain 0, orange; I, purple; II, blue; III, magenta; IV, yellow; V, pink; VI, green, 5.8S, brown, 5S, light green. The domain colors in the SSU are, 5′, blue; C, brown; 3′M, pink; and 3′m green. Fully detailed 2° structures of rRNAs, including base pairs and additional information, from *E. coli, T. thermophilus*, *H. marismortui, S. cerevisiae, D. melanogaster*, and *H. sapiens* are available at http://apollo.chemistry.gatech.edu/RibosomeGallery.

Our effort here is motivated in part by recent Cryo-EM structures of *D. melanogaster* and *H. sapiens* [6], which are extremely large, with highly complex secondary structures. In total, we have generated structure-based 2° structures for rRNAs of *E. coli* (Figures 1a & 1b), *T. thermophilus, H. marismortui* (LSU rRNA only), *S. cerevisiae* (Figures 1c & 1d), *D. melanogaster*, and *H. sapiens*. Previous *E. coli* [2], [7] and *S. cerevisiae* [8], [9] rRNA 2° structures, which lack the non-canonical central helix in the LSU rRNA (Helix 26a), and other non-canonical base pairs, have been presented. We previously described 2° structures of large subunit (LSU) rRNAs (23S/28S/5.8S/5S) of *E. coli, T. thermophilus, H. marismortui*, and *S. cerevisiae* [5].

## Methods

Atomic coordinates were obtained from the PDB. Base-pairing and base-stacking interactions were obtained from the library of RNA interactions (FR3D) [11] and confirmed by inspection and in-house code. The co-variation *E. coli* secondary structures of LSU and SSU rRNAs were downloaded from http://rna.ucsc.edu/rnacenter/ribosome_images.html, adjusted and extended with the program XRNA (http://rna.ucsc.edu/rnacenter/xrna/xrna.html), finalized with Adobe Illustrator, and written out as svg and png files. Secondary structures of all other species presented here were built from the *E. coli* template. We use historical representations as far as possible, except where conflicts arise with correct helical assignments or strand continuity.

*E. coli* 2° structures (Figure 1a & 1b) were determined from the x-ray structure of Cate [12] (PDB entries 3R8S, 4GD1, resolution 3.0 Å). *T. thermophilus* 2° structures were determined from the x-ray structure of Ramakrishnan [13] (PDB entries 2J00, 2J01, resolution 2.8 Å). *S. cerevisiae* 2° structures (Figure 1c & d) were determined from the x-ray structure of Yusupov [14] (PDB entries 3U5B, 3U5C, 3U5D, 3U5E, resolution 3 Å). *D. melanogaster* and *H. sapiens* 2° structures were determined from the cryo-EM structures of Beckmann [6] (PDB entries (3J38, 3J3C, 3J39, 3J3E for *D. melanogaster*, resolution 6 Å; PDB entries 3J3A, 3J3B, 3J3D, 3J3F, resolution 5 Å for *H. sapiens*).

## Results and Discussion

rRNA 2° structures can be determined by a variety of methods including co-variation [7], [15], [16], thermodynamic predictions [17] and by geometric analysis of molecular interactions within 3D structures [5]. We have re-derived a series of rRNA 2° structures from 3D structures, with the goal of improving clarity, accuracy, and utility. The primary disadvantage of the structural approach remains the small number of ribosomes with well-determined 3D structures. However, the number of ribosomes with available 3D structures is ever increasing [6], [8], [18]. The current numbers of available 3D structures make the geometric method a viable method for systematic determination of rRNA 2° structures.

Helices are the defining elements of RNA 2° structure [19], [20]. We identify helices by specific geometric and molecular interaction criteria [5]. In folded RNAs, a base is in one of two discrete states: paired or non-paired [21], [22]. A paired base is involved in 2° interactions, tertiary interactions, or both. Following Levitt [23], we define helices as base-paired nucleotides bounded by non-paired nucleotides. With 3D information, one can incorporate stacking information, and so we define helices as base pairs in the form of a continuous base-paired stack that is faithful to strand connectivity. A helix can contain bulges or other defects as long as they do not break the helical stack. Secondary interactions are base pairing interactions within helical regions, while tertiary interactions are pairing interactions other than those within helical regions. Each nucleotide belongs uniquely to no more than one helix. Non-canonical base pairs are not differentiated from canonical base pairs. Non-canonical base pairs that are internal to or that extend secondary helices are defined as secondary interactions.

**Figure 2:**
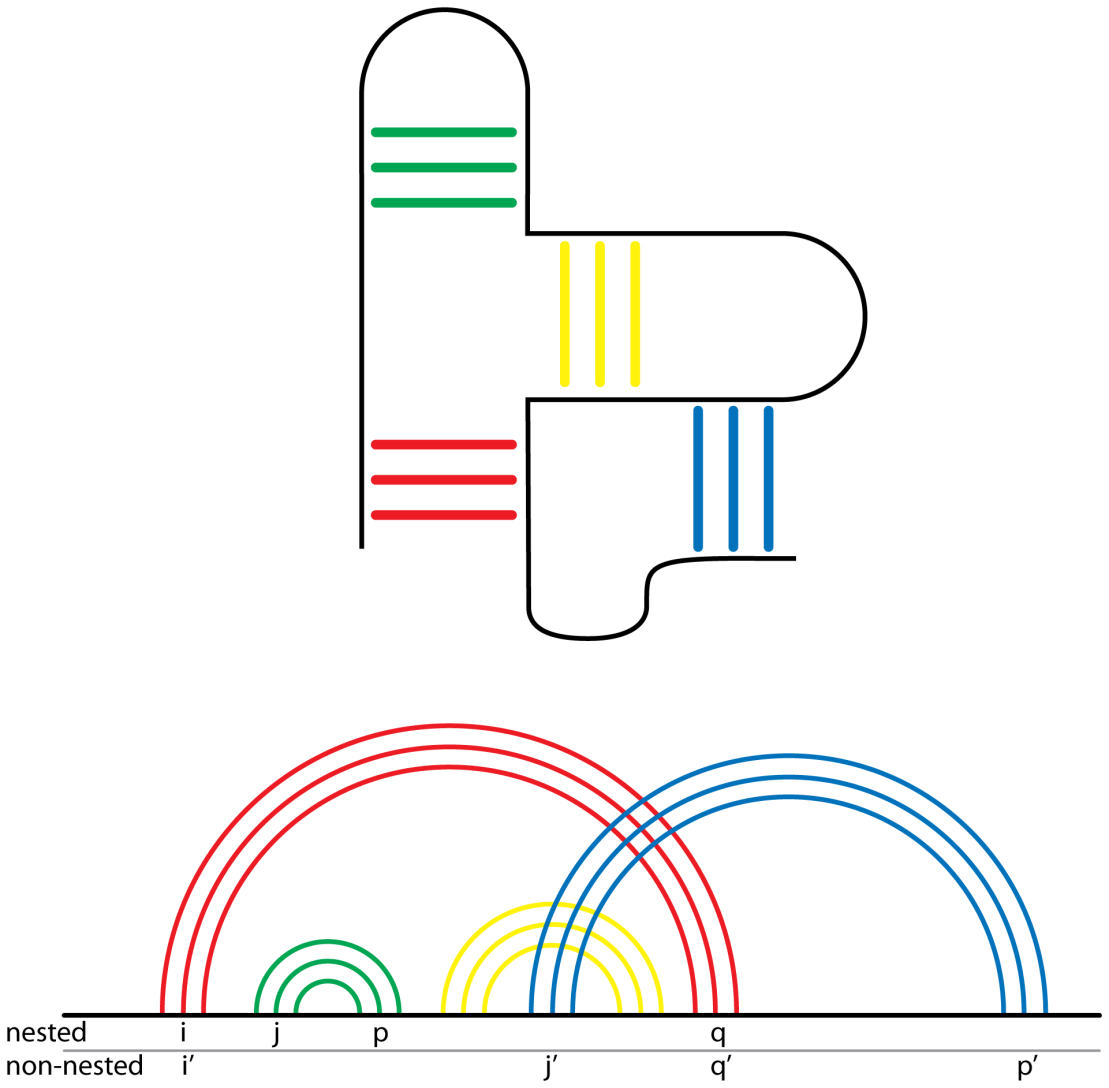
Nested and non-nested rRNA helices. A 2° structure with four helical regions is shown in the top panel. A topology diagram, illustrating the nesting concept, is shown in the bottom panel. The green and yellow helices are nested within the red helix, with base pairs (i,q) (red) and (j,p) (yellow or green) where i<j<p<q. The blue helix is non-nested within the other helices, with base pairs (i′,q′) (red) and (j′,p′) (blue) where i′<j′<q′<p′. The red, green and yellow helices are commonly considered to be 2° structural helices. The blue helix is non-nested and is considered to be a tertiary helix.

The basic helical definition of secondary structure [19] has been extended to differentiate helices that are nested from those that are non-nested [24]–[26], as illustrated in Figure 2. A structure is nested if it contains pairs (i,q) and (j,p) where i<j<p<q are locations in the primary structure. Helices between expansion elements observed in some eukaryotes (as in the 18S rRNAs of *S. cerevisiae, D. melanogaster*, and *H. sapiens*) are among the longest non-nested helices. Non-nested helices (kissing loops and pseudoknots) are commonly categorized as tertiary interactions [27], [28].

In our structure-based 2° structures, we followed the nest/non-nest definition of secondary and tertiary helices. Our approach extends and clarifies the definition of rRNA 2° structure to explicitly include *all* pairing interactions that confer thermodynamic stability to the folded RNA. The structural approach allows us to incorporate non-canonical base pairs on parity with Watson-Crick base pairs rather than by *post hoc* adjustment or symbolic notation.

**Figure 3:**
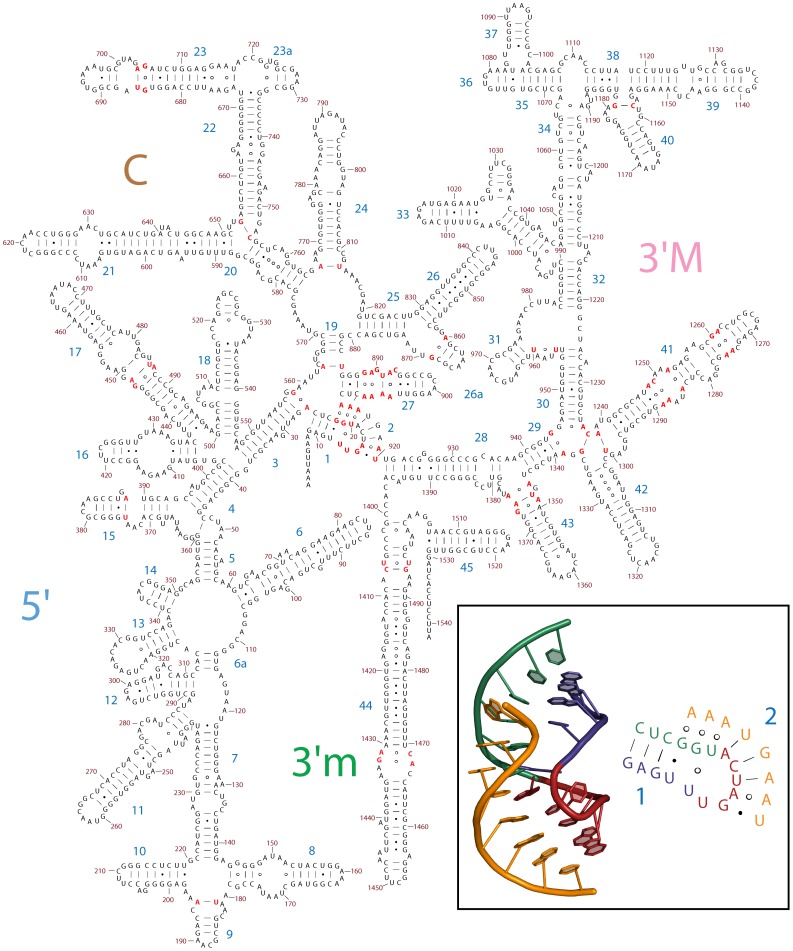
The 2° structure of the 16S rRNA of *E. coli*, based on three-dimensional structures. Regions where base-pairing interactions were modified relative to the co-variation 2° structure are highlighted in red. The inset shows the 2° and three-dimensional structures of the central pseudoknot (nucleotides 9–25 and 913–920). Nucleotides 9-13 are blue, nucleotides 14–19 are red, nucleotides 20–25 are green and nucleotides 913–920 are orange. The topology of the A915-U15-U20 triple is difficult to represent clearly in the 2° structure: A915 is base-paired with U15, which is base paired with U20 to form a base triple. This representation includes the sequence of the 16S rRNA and the helix and domain numbers.

For the central pseudoknot of the 16S rRNA [29], we treat helix 2 as a secondary element, even though it is non-nested, following the original Woese representation [15]. The central pseudoknot is conserved over all phylogeny [30] and is a key feature of the SSU that links all four domains. Central pseudoknot assembly appears to be a crucial, irreversible step of SSU maturation [31]. The co-variation 2° structure of the central pseudoknot is incomplete. We modified the traditional 2° structure of the central pseudoknot to include all base-paring interactions revealed by 3D structures. The central pseudoknot contains conserved triplets of bases U12-G22-A912 and U13-U20-A914. In our revised 2° structure, these base triples are presented as pairs of base pairs (Figure 3). The advantage of this representation is that one can easily infer that it is a pseudoknot and can directly discern all the pairing interactions of the pseudoknot. The representation used here was formulated by Brakier-Gingras and coworkers [32] and by Gregory and Dahlberg [33] using information from 3D crystal structures. Westhof and Lescoute correctly represent the central pseudoknot in their information-rich wiring diagrams [34]. Gutell recently revised the historical 2° structure of the 16S rRNA to adjust the central pseudoknot and incorporate many of the non-canonical base pairs [35]. Unlike other pseduoknots in the rRNA, this representation can be integrated into the historical 2° scheme without major rearrangement. The 3D based 2° structure of the 16S rRNA of *E. coli* with all canonical secondary and tertiary Watson-Crick interactions is shown in Figure S2.

## Conclusion

We have generated structure-based 2° structures for 23S/28S and 16S/18S rRNAs of *E. coli, T. thermophilus, S. cerevisiae*, *H. marismortui* (LSU only), *D. melanogaster*, and *H. sapiens*. We have mapped the 2° structures with a variety of data related to helices, domains, molecular interactions, phylogeny, and evolution. We provide high-resolution editable versions of all of these 2° structures (http://apollo.chemistry.gatech.edu/RibosomeGallery).

## Supporting Information

Figure S1 Schematic 2° structures, based on 3D structures, of rRNAs of a) *S. cerevisiae* LSU, and b) *S. cerevisiae* SSU. Major differences between these 2° structures and co-variation based 2° structures are highlighted in red: i) Helix 26a is shown as a helix instead of a single stranded loop; ii) the central pseudoknot is corrected to include all non-canonical base pairs; iii) rRNA is represented as far as possible as continuous strands; and iv) the secondary structure of all eukaryotic expansion segments is shown explicitly. The domain colors in the LSU are, Domain 0, orange; I, purple; II, blue; III, magenta; IV, yellow; V, pink; VI, green, 5.8S, brown, 5S, light green. The domain colors in the SSU are, 5′, blue; C, brown; 3′M, pink; and 3′m green.
(TIF)

Figure S2 The 2° structure of the 16S rRNA of *E. coli*. Nucleotides connected by lines in the 2° structure here are canonical Watson-Crick base-pairs in the 3D structure of the ribosome. The domain colors in the SSU are, 5′, blue; C, brown; 3′M, pink; and 3′m green.
(TIF)
