# Supplementary figures and images for: Secondary Structures of rRNAs from All Three Domains of Life
Source: PLoS One. 2014 Feb 5;9(2):e88222. doi: 10.1371/journal.pone.0088222 (PMC3914948; doi:10.1371/journal.pone.0088222)

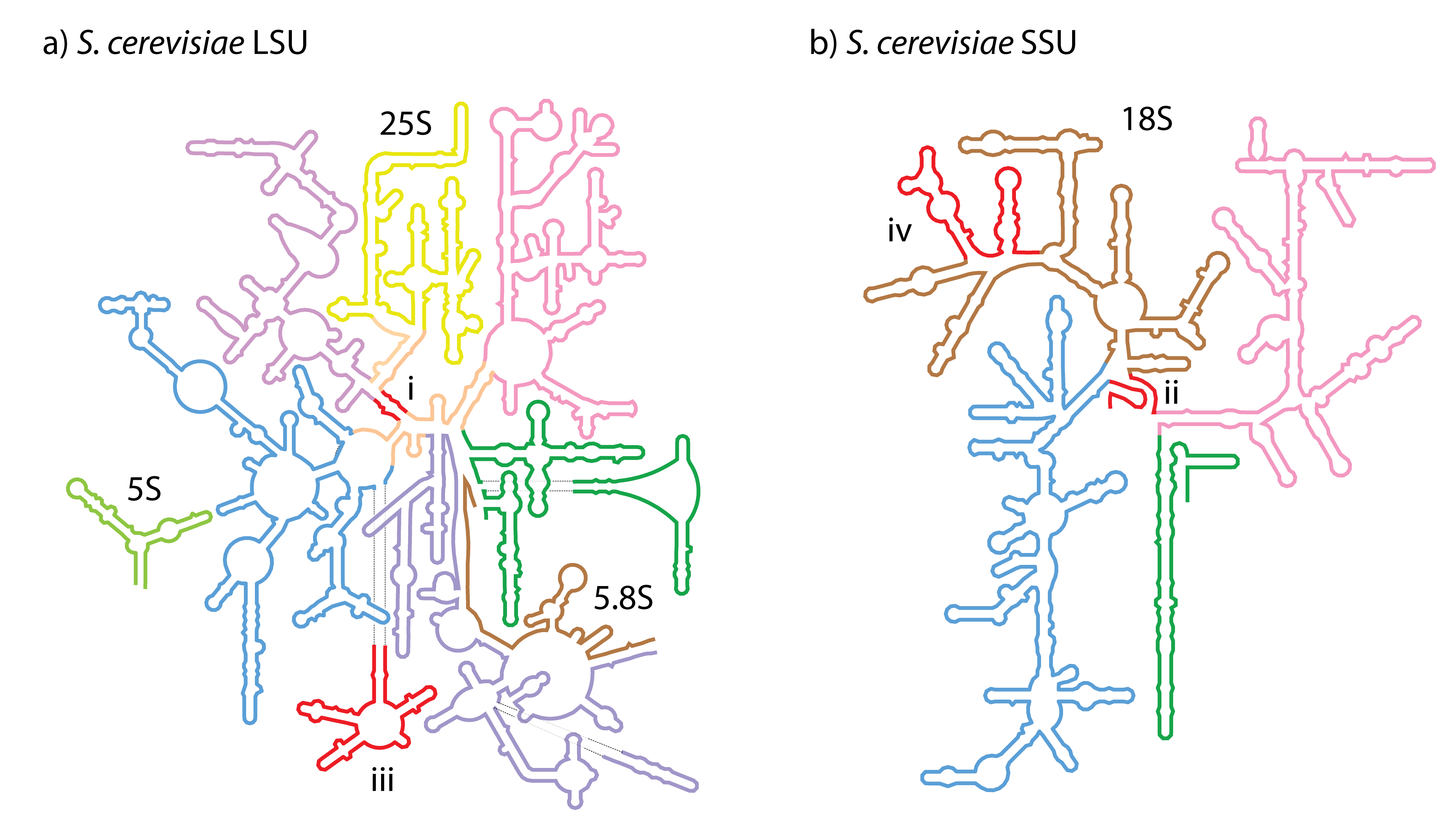

Supplement: Figure S1 — Schematic 2° structures, based on 3D structures, of rRNAs of a) S. cerevisiae LSU, and b) S. cerevisiae SSU. Major differences between these 2° structures and co-variation based 2° structures are highlighted in red: i) Helix 26a is shown as a helix instead of a single stranded loop; ii) the central pseudoknot is corrected to include all non-canonical base pairs; iii) rRNA is represented as far as possible as continuous strands; and iv) the secondary structure of all eukaryotic expansion segments is shown explicitly. The domain colors in the LSU are, Domain 0, orange; I, purple; II, blue; III, magenta; IV, yellow; V, pink; VI, green, 5.8S, brown, 5S, light green. The domain colors in the SSU are, 5′, blue; C, brown; 3′M, pink; and 3′m green. (TIF) [file pone.0088222.s001.tif]

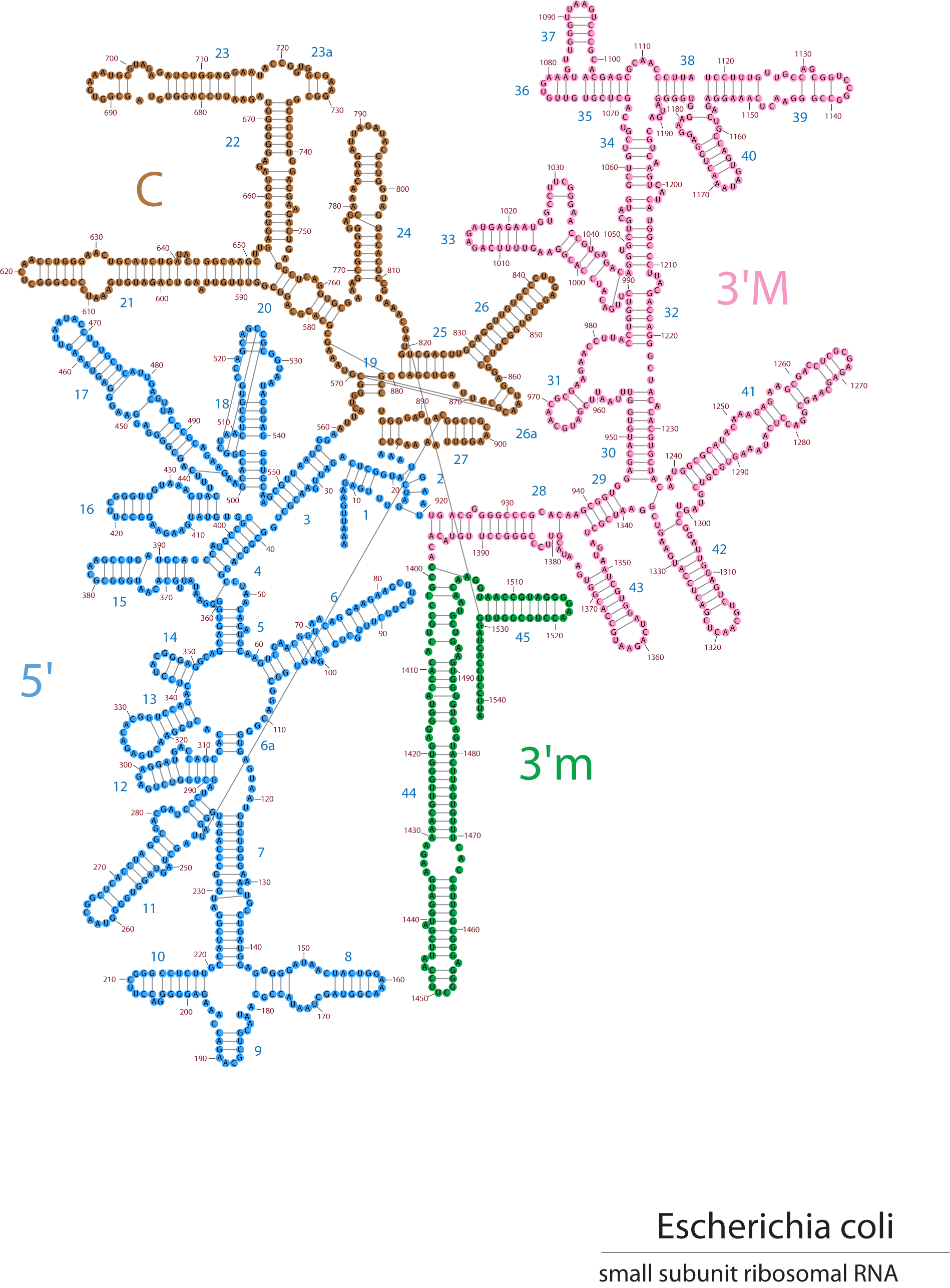

Supplement: Figure S2 — The 2° structure of the 16S rRNA of E. coli. Nucleotides connected by lines in the 2° structure here are canonical Watson-Crick base-pairs in the 3D structure of the ribosome. The domain colors in the SSU are, 5′, blue; C, brown; 3′M, pink; and 3′m green. (TIF) [file pone.0088222.s002.tif]
